# The Effects of the National Centralized Drug Purchasing Pilot Program on Nucleos(t)ide Analogs in Shenzhen City: An Interrupted Time Series Analysis

**DOI:** 10.3389/fpubh.2021.718013

**Published:** 2021-10-25

**Authors:** Xiaotong Wen, Shicheng Yin, Lanyue Cui, Lining Mao, Zhaoyu Lin, Zilalai Yaermaimaiti, Xin Geng, Yingxia Li, Ying Yang, Dan Cui, Zongfu Mao

**Affiliations:** ^1^Department of Social Medicine and Health Service Management, School of Health Sciences, Wuhan University, Wuhan, China; ^2^Department of Global Health, Global Health Institute, Wuhan University, Wuhan, China; ^3^Department of Gynecologic Oncology, Sun Yat-sen University Cancer Center, Guangzhou, China

**Keywords:** pharmaceutical expenditures, interrupted time series analysis, policy effect, usage volume, daily cost, medicine procurement policy, nucleos(t)ide analogs, hepatitis B virus

## Abstract

**Objectives:** To assess the effects of the National Centralized Drug Purchasing Pilot Program on nucleos(t)ide analogs (NAs) in Shenzhen city.

**Methods:** Drugs procurement records in medical institutions were analyzed covering the period from January 2018 to December 2019. An interrupted time series (ITS) analysis was used to evaluate the impact of the “4+7” pilot policy on NAs in Shenzhen city. The outcome measures were usage volume, expenditures, daily cost, and distribution structure of NAs.

**Findings:** After the introduction of the “4+7” pilot policy, the defined daily doses (DDDs) of NA drugs increased by 76.48%, the expenditures and defined daily dose cost (DDDc) of NAs decreased by 45.43 and 69.08%, respectively. The proportion of winning products in Entecavir and Tenofovir Fumarate DDDs was increased by 64.21 and 19.20%, respectively. The post-intervention period witnessed a significant increase in the regression level for NAs DDDs (level coefficient: β_2_ = 631.87, *p* < 0.05). The expenditures (trend coefficient: β_3_ = 392.24, *p* < 0.05) and DDDc (level coefficient: β_2_ = −6.17, *p* < 0.001; trend coefficient: β_3_ = −0.21, *p* < 0.05) of NAs showed decreasing trend in the post-intervention period. The expenditures of original products and generic products both showed a decreasing trend in the post-intervention period (trend coefficient: β_3_ = −372.78, *p* < 0.05, trend coefficient: β_3_ = −130.78, *p* < 0.05, respectively). The DDDc of original products in the policy-related varieties was a significant decrease in the regression slope and level (level coefficient: β_2_ = −2.18, *p* < 0.05; trend coefficient: β_3_ = −0.32, *p* < 0.01).

**Conclusion:** After the implementation of the“4+7” policy, the DDDc of NAs decreased, the accessibility of policy-related drugs was improved, and the usage of generic medicine was promoted.

## Introduction

The global pharmaceutical spending is set to exceed $1.5 trillion by 2023 growing at a 3–6% compound annual growth rate over the next 5 years, as a new report by the Quintiles IMS Holdings, Inc (IQVIA) Institute, a healthcare industry consulting group (1). Many countriesare facing the challenge of ever-increasing pharmaceutical expenditures all over the world. Using the IQVIA National Sales Perspectives database, total pharmaceutical expenditures in the United States were $535.3 billion in 2020, which grew 4.9% compared to 2019 (2). Medicine spending in Japan totaled $86 billion in 2018. A study using the Korean National Health Insurance claims database revealed that total pharmaceutical expenditures increased from $11.3 billion in 2010 to $17.4 billion in 2019 (3). Medical expenditures as a proportion of gross domestic product (GDP) also increased steadily from 5.9% in 2010 to 8.0% in 2019 (3). Pharmaceutical expenditure in lower-middle-income countries can be up to 70% of total healthcare expenditure, much of which is out-of-pocket (OOP) (4). Medicines account for 20–60% of health spending in developing and transitional countries. Up to 90% of the population in developing countries face high OOP expenditures when purchasing medicines, making medicines the important family expenditure item. As a result, pharmaceutical spending is unaffordable for large sections of the global population (5).

In China, rapidly increasing pharmaceutical expenditures threaten the sustainability of the Chinese health insurance system (6). Pharmaceutical spending in China reached $137 billion in 2018 and is expected to reach $140–170 billion by 2023, but its growth is likely to slow to 3–6%. Furthermore, pharmaceutical expenditures account for a large proportion of total health expenditures (THE) (7, 8). In 2016, pharmaceutical expenditures comprised 35.8% in THE in China, which was much higher than (29.5%) the highest of Organization for Economic Cooperation and Development (OECD) nations (9). Pharmaceutical expenditure in lower-middle-income countries can be up to 70% of THE, much of which is OOP, compared to up to 17% in higher-income countries (4, 10). Pharmaceutical expenditures depended on drug prices and drug volume, which were corresponding to the supply and demand sides of drugs, respectively (11). As a result, the control of drug prices was a direct measure associated with the supply side to reduce pharmaceutical expenditures.

Reduction of drug prices has been enforced in many countries given that they can bring about an immediate reduction in pharmaceutical spending (12–15). Countries across the world are implementing various pharmaceutical pricing policies and procedures to cope with increasing drug prices (16). We have seen such practices work well in other countries. This usually applies to multiple sourced medicines, e.g., in the Netherlands, their procurement practices resulted in very low prices (i.e., only 2% of pre-patent loss prices for generic omeprazole and generic simvastatin over time) (17). Low prices have also been obtained for oral cancer medicines across Europe, and we are now seeing these for biosimilars (18, 19). Reference pricing has been shown to reduce drug spending in Europe and has been adopted by some employers and labor unions in the United States (20). Policymakers save money through generic drugs in the United States. They usually streamline the generic drug approval process and require generic prescribing and substitution where such policies are not yet in place (21). India has been regulating the prices of drugs included in its national list of essential medicines (22).

In China, the General Office of the State Council issued the National Centralized Drug Purchasing (23) and Using Pilot Program on January 1, 2019, to intensify the reform of the medical and healthcare system, improve the mechanism of the drug prices formation. In this pilot program, 11 cities were selected as pilot cities to carry out drug volume-based purchasing, i.e., four municipalities (Beijing, Tianjin, Shanghai, and Chongqing) and seven sub-provincial cities (Shenyang, Dalian, Xiamen, Guangzhou, Shenzhen, Chengdu, and Xi'an). Therefore, this pilot program was also known as the “4+7” pilot policy (24).

Pharmaceutical expenditure is increasingly steady by payers in view of its rapid growth, outstripping growth in other components of healthcare. To significantly reduce drug prices and lessen the heavy burden of expenses of patients for medicine, the program selected pilot varieties of drugs, such as original drugs, generic drugs, and corresponding reference preparation. The generic drugs have been evaluated for consistency in quality and efficacy. Pricing regulations for generics were introduced in Austria (25). The price of the third generic has to be at least 60% below the single-sourced prices to be reimbursed (26, 27). As a consequence, market forces act to obtain lower prices for generics. Multiple reforms and initiatives can help control expenditure on existing drugs by encouraging the increased prescribing of generics at low prices (28).

First of all, the program formulates content from the national level, such as basic policies, scope, and requirements. Secondly, the program organizes 11 pilot cities to form a purchasing alliance (29). In this program, the public medical institutions in 11 pilot cities are the main purchasing entities. The program selected pilot varieties of drugs, such as original drugs, generic drugs, and corresponding reference preparation. The generic drugs have been evaluated for consistency in quality and efficacy. Based on the purchasing quantity submitted by the public medical institutions in the purchasing alliance, according to 60–70% of the total annual drug use of all public medical institutions in the purchasing alliance, it conducted a mechanism for drug purchasing which linked volume and price. The program determined the purchase price of drugs according to the specific quantity through bidding or negotiating. The drug supply enterprises reduced drug prices for the purpose of obtaining a larger market. From another perspective, it was a form of group purchasing in the pharmaceuticals industry with a deeply discounted.

China has the heaviest hepatitis B virus (HBV) infection burden and the greatest number of people living with HBV infections in worldwide (30). There were an estimated 100 million people chronically infected with HBV in China, accounting for almost 39% of the global total HBV infections (31). As a result, HBV has long been a public health threat in China. One Chinese hepatitis epidemiological serosurvey found that hepatitis B endemicity in China shifted from high to intermediate after the implementation of effective nationwide vaccination programs, but it also suggested no significant change in prevalence among adults aged 20–59 years (32). Patients with chronic hepatitis B (CHB) are at high risk of developing serious complications, such as cirrhosis of the liver (LC), liver failure, and hepatocellular carcinoma (HCC) if they do not have timely intervention (33, 34). A study in patients with CHB revealed that up to 25% of patients with CHB eventually develop into HCC (35). In China, the high prevalence of HCC is mainly due to HBV infection (36, 37), and approximately 80% of Chinese HCCs patients are caused by HBV infection (38). As a result of the World Cancer Report in 2014, half of the new cases and deaths cases of HCC patients in the global were from China (39).

Some studies suggested that effective antiviral therapy can reduce the incidence of HCC in patients with hepatitis B (40–42). Compared with the control group untreated patients, 1,446 patients with CHB who received antiviral treatment, the cumulative incidence rates of HCC in patients at 3 and 5 years were reduced in the treatment group. Some studies in Japan showed similar results (43–45). From the perspective of health economics, antiviral therapy for patients with HBV has a good cost-effectiveness ratio. That is to say, if no active measures are taken to treat CHB now, more resources will be needed to treat LC and HCC caused by hepatitis B in the future (46). A study in 12 cities in China has shown that HBV-related diseases have caused a substantial financial burden to Chinese patients, their families, and society as a whole (47). The results revealed that annual direct medical costs of HBV-related diseases among hospitalized patients have reached $4197.9, which accounted for 206.5% of the income of patients (47). Drug costs accounted for a large proportion of direct medical costs. Antiviral treatment was the key to the treatment of patients with CHB, which could improve the quality of life of patients and prolong the survival time. Nucleos(t)ide analog (NA) drugs were important antiviral therapy drugs for patients with CHB (48). But only 11% of 30 million patients with CHB received standardized antiviral treatment in China (31) for poor medicines affordability.

Although NA drugs have been included in the National Medical Insurance Drug Catalog, specific problems, such as connecting with local medical insurance, outpatient reimbursable policy, and reducing the proportion of patients paying their expenses, can still be solved to bring benefits to the majority of patients with CHB. Previous studies have shown that health insurance policy could promote the utilization of antiviral drugs in patients with CHB (49). The direct medical costs of outpatients were higher since health insurance policies often provide a higher proportion of reimbursement for inpatients than for outpatients. In the long run, the insurance system should be improved to narrow the gap between different medical insurance policies, and it is important to gradually enlarge the outpatient coverage and improve the utilization of antiviral drugs. The Outpatient Medical Insurance Fund Policy provided monthly quota payments for patients in Guangzhou. Both urban and rural residents were paid with patients with CHB 480 RMB per month by this Fund from December 25, 2018. Until March 15, 2021, insured patients with CHB were paid 10,000 RMB every year by The Outpatient Medical Insurance Fund in Shenzhen. Direct medical costs were including the following categories: (1) laboratory tests and imaging examinations, (2) antiviral therapy, (3) other treatment of HBV infection such as hepatoprotective drugs and traditional Chinese medication, and (4) other costs including registration and consultation costs, etc. (50). If the price of NA drugs decrease, more patients may get antiviral treatment on time, and the economic burden will be lighter.

In the “4+7” pilot program, a total of 25 drugs won the bidding, only one company is selected for each variety, and the purchasing cycle is 12 months. In the purchasing cycle, the price of drugs stays the same when the agreed purchase volume is completed ahead of the purchasing cycle. In other words, the excess of the agreed purchase volume will still be purchased with the bid price until the purchase cycle expires. The price of 25 successful bid drugs decreased, whose average price drop was 52% and the highest price drop was 96%. The massive price cuts dramatically impacted overall drug expenditures. The NA represents the treatment option for the majority of patients with CHB (48), such as Entecavir, Tenofovir Fumarate, Lamivudine, Adefovir dipivoxil, and Telbivudine. Entecavir and Tenofovir Fumarate are guanine NAs for the treatment of HIV infection, which were equally recommended by the Guidelines as the first-line antiviral treatments for CHB at present (51). In the first round of the “4+7” pilot program, a total of 25 drugs won the bidding. Entecavir and Tenofovir Fumarate were included. Entecavir and Tenofovir Fumarate are first-line anti-HBV drugs recommended by the Guidelines for CHB. The price of the original drug Entecavir produced by Bristol-Myers Squibb was ¥175.68 per box (seven tablets/box). As a drug winning the bidding, the price of generic drug Entecavir was ¥17.36 per box (28 tablets/box) which was produced by Chia Tai Tianqing. In other words, the annual cost of the anti-viral medication will be reduced from about ¥9,000 to about ¥200, and the economic burden of patients will be lighter. The price of generic drug Tenofovir Fumarate decreased by 96% compared with the original drug Tenofovir Fumarate.

The objectives of this study were to quantitatively evaluate the impacts of the “4+7” pilot policy on the volume, expenditures, daily cost, and distribution structure of NAs. Our hypothesis was that the “4+7” pilot policy would sufficiently control the growth of total drug spending of the NAs class. The expenditure of both drugs winning the bidding and alternative drugs was expected to decrease. Thus, the overall expenditure would also decrease. That is to say, price cuts would mainly affect the growth of total drug expenditure. To prove this, we first analyzed whether the “4+7” pilot policy would lead to a decrease in the overall expenditure of the NAs class. Second, we tried to do a subgroup analysis for drug policy-related varieties and alternative drugs, winning and non-winning drugs, and generic and original drugs. Finally, we examined the increasing trend of the volume and the expenditure of NAs.

## Materials and Methods

### Data Sources

Data on products purchased between January 2018 and December 2019 were extracted from the Centralized Drug Procurement Survey in Shenzhen, which was 1 of 11 pilot cities to carry out drug volume-based purchasing. The project administration and supervision were the School of Health Sciences and the Global Health Institute of Wuhan University. The project collected monthly drug purchase order records from January 2018 to December 2019 from each medical institution. There were 963,127 monthly aggregated drugs purchase order records in the database. According to the generic name of drugs, there were 1,079 drug varieties in the order records database. The drug purchase order records involved 346 medical institutions and 857 pharmaceutical manufacturers. Each drug purchase order record included the code of drug, generic name, dosage form, specification parameter, procurement unit, price per unit, conversion coefficient, pharmaceutical manufacturer, purchase expenditures, medical institutions, type of medical institution, purchase date, purchase volume, and purchase expenditures.

In this study, we analyzed that NA classes which included Entecavir, Tenofovir Fumarate, Lamivudine, Adefovir dipivoxil, and Telbivudine. Then, we made a classification in Entecavir, Tenofovir Fumarate about products with winning the bidding or not, and original and generic drugs.

### Outcome Measures

The primary outcomes were volume, expenditures, and daily costs of drugs. The volume of drugs was measured by defined daily dose (DDD), a standard measure of the volume of each product procured to calculate and compare drug consumptions. Defined daily dose was limited within the routine dose of the main adaptation syndrome in adults. The DDDs used in this paper were the recommended daily amounts of each study medication based on dosage regimens recommended in the instructions of manufacturers, as approved by the China Food and Drug Administration. The maintenance dose of Entecavir, Tenofovir Fumarate, Lamivudine, Adefovir dipivoxil, and Telbivudine in this study were 0.5, 300, 100, 10, and 600 mg, respectively. According to the WHO Collaborative Centre for Drug Statistics Methodology, DDD equivalence per package (DPP) of medicines was calculated using the formula:


$$\begin{array}{l} DPP\;=(conversion\;coefficient\times specification\;parameter)/\\ \text{​​​​​​​​​​​​​​​​​​​​​​​​​​​​​​​​​ ​}DDD.\; \end{array}$$

The total volume for each group drug (DDDs) was calculated by summed DPPs of all packaged products with the same category. Defined daily dose of each group drug was calculated using the formula:


$$DDD_{s}\;=\;\sum_{i=1}^{n}\left(DPP_{i}\times N_{i}\right)$$

In the formula, *N*_*i*_ represents the number of packages of a certain product (*i*).

We calculated the DDDs for comparing drug consumptions among different drugs produced by different pharmaceutical manufacturers. Then we compared drug consumptions among different generic names, such as Entecavir, Tenofovir Fumarate, Lamivudine, Adefovir dipivoxil, and Telbivudine. We also compared drug consumptions between winning the bidding or not and original and generic drugs.

The expenditures of drugs were measured by the amount of drug purchase order records which were calculated by Chinese yuan. We also calculated the expenditures among different drugs produced by different pharmaceutical manufacturers. Then we added expenditures among different generic names in NAs. We also compared drug expenditures between winning and non-winning drugs and original and generic drugs.

The daily cost of drugs was measured by defined daily dose cost (DDDc), a standard measure of the procurement cost of each product (52). In this study, DDDc was calculated using the formula:


$$DDD_{c}\;=\;Expenditures(yuan)/DDD_{s}\;$$

### Statistical Analysis

First of all, on DDDs, expenditures, and DDDc of hepatitis B antiviral NAs, we used descriptive statistical analysis to compare two periods which are pre-intervention period (from March 2018 to December 2018 before implementation of the “4+7” policy) and post-intervention period (from March 2019 to December 2019 after implementation of the “4+7” policy), the calculated growth rate between the two periods.

Second, we compared the constituent ratio of DDDs, expenditures, and DDDc of hepatitis B antiviral NAs between the two periods and analyzed the variation of constituent ratio.

Finally, we conducted interrupted time series (ITS) analysis with segmented regression analysis of each study medication to assess the change in DDDs, expenditures, and DDDc associated with the implementation of the “4+7” policy. Interrupted time series is the best and most commonly used approach for evaluating the longitudinal effects of interventions occurring at a fixed point in time, such as the date of implementation of policy. Many researchers consider ITS analysis as the strongest quasi-experimental design to evaluate the effects of interventions (53).

In this article, ITS analysis was utilized to evaluate the impact of the “4+7” policy on the DDDs, expenditures, and DDDc. The date of implementation of the “4+7” policy in March 2019 was regarded as the intervention time point for ITS analyses. Therefore, two segments with one interruption point were constructed, one is the pre-intervention period and another is the post-intervention period. The following model was used for the analysis:


$$\begin{array}{l} {\text{Y}}_{\text{it}}\text{=}\beta_{\text{0}}\text{ + }\beta_{\text{1}}{}^{*}Time_{t}+\beta_{2}{}^{*}Intervention_{t}\\ \;+\beta_{3}{}^{*}Time\_after\_Intervention\;+\;\varepsilon_{it} \end{array}$$

*Y*_*it*_ is the independent outcome variable (DDDs, expenditures, or DDDc). β_0_ estimates the level of the outcome at the beginning of the observation period. β_0_ is a constant. β_1_ estimates the linear trend during the pre-intervention period. Time_t_ is a continuous variable defined as the number of periods at time *t*. β_2_ coded as Intervention_t_ = 0 is pre-intervention and Intervention_t_ = 1 is post-intervention, which estimates the change in the outcome immediately following the implementation of the “4+7” policy. β_3_ estimates the change in trend in the outcome during the post-intervention period. Time_after_Intervention represents the number of periods after the intervention at time t; for the time before the intervention, Time_after_Intervention = 0. β_2_ and β_3_ are the changes in the intercept and slope, respectively. ε_it_ is an estimate of the random error at Time_t_. We set the time point immediately following the implementation of the “4+7” policy. The Durbin-Watson statistic was performed to test for a serial autocorrelation of error terms in the regression models. This has involved testing for serial correlation by assuming a first-order autoregressive correlation structure. We performed the ITS analysis using Stata 16.0 (Stata Corporation, College Station, TX, USA).

### Ethical Statements

This study was approved by the institutional review board of Wuhan University. Patients or the public were not involved in this study.

## Results

### Descriptive Analysis of Changes in Volume, Expenditures, and DDDc

A total of five NA drugs (by generic name) were included in this study. Entecavir and Tenofovir Fumarate were the “4+7” policy-related variety drugs. Lamivudine, Adefovir dipivoxil, and Telbivudine were alternative varieties of drugs (Table 1). Descriptive statistics analysis was used to compare two periods which are the pre-intervention period (from March 2018 to December 2018) and the post-intervention period (from March 2019 to December 2019), the calculated growth rate between the two periods. As shown in Table 2, the DDDs of policy-related variety drugs were increased by 95.60% and the expenditures and DDDc of policy-related drugs were decreased by 47.69 and 73.26%, respectively. The DDDs of NA drugs increased 76.48%, the expenditures and DDDc of NAs decreased by 45.43 and 69.08%, respectively. The DDDs of Entecavir were increased by 80.45% and the expenditures and DDDc of Entecavir were decreased by 48.57 and 71.50%, respectively. The DDDs of Tenofovir Fumarate were increased by 156.58% and the expenditures and DDDc of Tenofovir Fumarate were decreased by 45.52 and 78.77%, respectively. The DDDs of Lamivudine, Adefovir dipivoxil, and Telbivudine were decreased by 47.08, 62.86, and 26.09%, respectively.

**Table 1:** The information of all the NAs medicines.

**Category**	**Generic name**	**YPID**	**Winning/Non-winning**	**Original/Generic**	**Dosage forms**	**Strengths (mg)**
**Policy-related varieties**						
	Entecavir	G0182064	Winning products	Generic products	Dispersible tablets	0.5
	Entecavir	M0001502	Non-winning products	Original products	Film-coated tablets	0.5
	Entecavir	M0001501	Non-winning products	Generic products	Hard capsule	0.5
	Tenofovir Fumarate	G0003509	Winning products	Generic products	Film-coated tablets	300
	Tenofovir Fumarate	M0000764	Non-winning products	Original products	Film-coated tablets	300
**Alternative varieties**						
	Lamivudine	M0002039	–	–	Film-coated tablets	100
	Lamivudine	M0002040	–	–	Tablets	100
	Telbivudine	M0002776	–	–	Film-coated tablets	600
	Adefovir dipivoxil	M0001040	–	–	Tablets	10
	Adefovir dipivoxil	M0001042	–	–	Hard capsule	10

*NAs, nucleos(t)ide analogs*.

**Table 2:** Descriptive analysis of hepatitis B antiviral NAs in Shenzhen.

**Categories**	**DDDs (ten thousand)**			**Expenditures (ten thousand yuan)**			**DDDc (yuan)**		
	**Mar.–Dec. 2018**	**Mar.–Dec. 2019**	**Growth rate (%)**	**Mar.–Dec. 2018**	**Mar.–Dec. 2019**	**Growth rate (%)**	**Mar.–Dec. 2018**	**Mar.–Dec. 2019**	**Growth rate (%)**
**Policy-related varieties**	**947.69**	**1853.69**	**95.60**	**10622.83**	**5557.13**	**−47.69**	**11.21**	**3.00**	**−73.26**
Entecavir	759.06	1369.69	80.45	7541.81	3878.47	−48.57	9.94	2.83	−71.50
Tenofovir Fumarate	188.63	484.00	156.58	3081.02	1678.66	−45.52	16.33	3.47	−78.77
**Alternative varieties**	**152.30**	**87.62**	**−42.47**	**1776.19**	**1209.14**	**−31.93**	**11.66**	**13.80**	**18.33**
Lamivudine	24.06	12.74	−47.08	216.43	116.90	−45.99	8.99	9.18	2.06
Adefovir dipivoxil	54.11	20.10	−62.86	255.02	128.17	−49.74	4.71	6.38	35.31
Telbivudine	74.13	54.79	−26.09	1304.74	964.07	−26.11	17.60	17.60	−0.02
**NAs**	**1100.00**	**1941.31**	**76.48**	**12399.02**	**6766.27**	**−45.43**	**11.27**	**3.49**	**−69.08**

*NAs, nucleos(t)ide analogs; DDDs, defined daily doses; DDDc, defined daily dose cost*.

Table 3 showed the constituent ratio of DDDs, expenditures, and DDDc of hepatitis B antiviral NAs between the pre-intervention and post-intervention periods. Before the implementation of the “4+7” policy, the proportion of Entecavir in NAs DDDs was 69.01%, and the proportion of Tenofovir Fumarate was 17.15%. After the implementation of the “4+7” policy, both of the proportions of Entecavir and Tenofovir Fumarate in NAs DDDs were increased. Especially, Tenofovir Fumarate was increased by 7.78% points on DDDs. The proportion of policy-related variety drugs on DDDs was increased by 9.33% points in the post-intervention period, and the proportion of policy-related drugs on expenditures was decreased by 3.54 percentage points. After the implementation of the “4+7” policy, the proportion of Telbivudine in NAs DDDs was decreased, but the proportion of expenditures was increased by 3.73 points.

**Table 3:** Descriptive analysis of hepatitis B antiviral NAs constituent ratio in Shenzhen.

**Categories**	**The constituent ratio of DDDs (%)**			**The constituent ratio of expenditures (%)**		
	**Mar.–Dec. 2018**	**Mar.–Dec. 2019**	**Variation**	**Mar.–Dec. 2018**	**Mar.–Dec. 2019**	**Variation**
**Policy-related varieties**	**86.15**	**95.49**	**9.33**	**85.67**	**82.13**	**−3.54**
Entecavir	69.01	70.55	1.55	60.83	57.32	−3.51
Tenofovir Fumarate	17.15	24.93	7.78	24.85	24.81	−0.04
**Alternative varieties**	**13.85**	**4.51**	**−9.33**	**14.33**	**17.87**	**3.54**
Lamivudine	2.19	0.66	−1.53	1.75	1.73	−0.02
Adefovir dipivoxil	4.92	1.04	−3.88	2.06	1.89	−0.16
Telbivudine	6.74	2.82	−3.92	10.52	14.25	3.73
**NAs**	**100.00**	**100.00**	**0.00**	**100.00**	**100.00**	**0.00**

*NAs, nucleos(t)ide analogs; DDDs, defined daily doses*.

Entecavir and Tenofovir Fumarate, which were “4+7” policy-related variety drugs, were divided into winning products and non-winning products and original and generic products, respectively. Table 4 demonstrated that the DDDs of winning products for Entecavir, which were produced by Chia Tai Tianqing, were increased from 0 to 1190.31 ten thousand. The DDDc of winning products for Entecavir was 0.62 yuan. The DDDs of winning products for Tenofovir Fumarate, which were produced by Brilliant Pharmaceutical, were increased from 0 to 355.96 ten thousand. The DDDc of winning products for Tenofovir Fumarate was ¥0.59.

**Table 4:** Descriptive analysis of hepatitis B antiviral NAs policy-related drugs in Shenzhen.

**Categories**	**DDDs (ten thousand)**			**Expenditures (ten thousand yuan)**			**DDDc (yuan)**		
	**Mar.–Dec. 2018**	**Mar.–Dec. 2019**	**Growth rate (%)**	**Mar.–Dec. 2018**	**Mar.–Dec. 2019**	**Growth rate (%)**	**Mar.–Dec. 2018**	**Mar.–Dec. 2019**	**Growth rate (%)**
Entecavir	759.06	1369.69	80.45	7541.81	3878.47	−48.57	9.94	2.83	−71.50
Winning products	0.00	1190.31	–	0.00	737.99	–	–	0.62	–
Non-winning products	759.06	179.38	−76.37	7541.81	3140.48	−58.36	9.94	17.51	76.21
Tenofovir Fumarate	188.63	484.00	156.58	3081.02	1678.66	−45.52	16.33	3.47	−78.77
Winning products	0.00	355.96	–	0.00	210.25	–	–	0.59	–
Non-winning products	188.63	128.04	−32.12	3081.02	1468.41	−52.34	16.33	11.47	−29.79
**Policy-related varieties**	**947.69**	**1853.69**	**95.60**	**10622.83**	**5557.13**	**−47.69**	**11.21**	**3.00**	**−73.26**
Winning products	0.00	1546.27	–	0.00	948.24	–	–	0.61	–
Non-winning products	947.69	307.42	−67.56	10622.83	4608.88	−56.61	11.21	14.99	33.75
Entecavir	759.06	1369.69	80.45	7541.81	3878.47	−48.57	9.94	2.83	−71.50
Original products	216.42	144.54	−33.21	5425.50	3004.59	−44.62	25.07	20.79	−17.08
Generic products	542.64	1225.15	125.78	2116.30	873.88	−58.71	3.90	0.71	−81.71
Tenofovir Fumarate	188.63	484.00	156.58	3081.02	1678.66	−45.52	16.33	3.47	−78.77
Original products	188.63	128.04	−32.12	3081.02	1468.41	−52.34	16.33	11.47	−29.79
Generic products	0.00	355.96	–	0.00	210.25	–	–	0.59	–
**Policy-related varieties**	**947.69**	**1853.69**	**95.60**	**10622.83**	**5557.13**	**−47.69**	**11.21**	**3.00**	**−73.26**
Original products	405.05	272.58	−32.70	8506.53	4473.00	−47.42	21.00	16.41	−21.86
Generic products	542.64	1581.11	191.37	2116.30	1084.13	−48.77	3.90	0.69	−82.42

*NAs, nucleos(t)ide analogs; DDDs, defined daily doses; DDDc, defined daily dose cost*.

Table 4 also demonstrated that the DDDs of generic products in policy-related varieties were increased by 191.37%. The expenditures and DDDc of generic products in policy-related varieties were decreased by 48.77 and 82.42%, respectively. The DDDs of generic products in Entecavir were increased by 125.78%. The expenditures and DDDc of generic products in Entecavir were decreased by 58.71 and 81.71%, respectively.

Table 5 revealed the constituent ratio of DDDs, expenditures, and DDDc of winning products and non-winning products, original products, and generic products between pre-intervention and post-intervention periods. Before the implementation of the “4+7” policy, the proportion of winning products in Entecavir and Tenofovir Fumarate DDDs both was 0.00%. After the implementation of the “4+7” policy, the proportion of winning products in Entecavir and Tenofovir Fumarate DDDs was increased 64.21 and 19.20%, respectively. The proportion of generic products in policy-related varieties was 57.26% in the pre-intervention. The proportion of generic products in Entecavir and Tenofovir Fumarate DDDs was increased by 8.83 and 19.20%, respectively.

**Table 5:** Descriptive analysis of hepatitis B antiviral NAs policy-related constituent ratio drugs in Shenzhen.

**Categories**	**The constituent ratio of DDDs (%)**			**The constituent ratio of expenditures (%)**		
	**Mar.–Dec. 2018**	**Mar.–Dec. 2019**	**Variation**	**Mar.–Dec. 2018**	**Mar.–Dec. 2019**	**Variation**
Entecavir	**80.10**	**73.89**	**−6.21**	**71.00**	**69.79**	**−1.20**
Winning products	0.00	64.21	64.21	0.00	13.28	13.28
Non-winning products	80.10	9.68	−70.42	71.00	56.51	−14.48
Tenofovir Fumarate	**19.90**	**26.11**	**6.21**	**29.00**	**30.21**	**1.20**
Winning products	0.00	19.20	19.20	0.00	3.78	3.78
Non-winning products	19.90	6.91	−13.00	29.00	26.42	−2.58
**Policy-related varieties**	**100.00**	**100.00**	**0.00**	**100.00**	**100.00**	**0.00**
Winning products	0.00	83.42	83.42	0.00	17.06	17.06
Non-winning products	100.00	16.58	−83.42	100.00	82.94	−17.06
Entecavir	**80.10**	**73.89**	**−6.21**	**71.00**	**69.79**	**−1.20**
Original products	22.84	7.80	−15.04	51.07	54.07	2.99
Generic products	57.26	66.09	8.83	19.92	15.73	−4.20
Tenofovir Fumarate	**19.90**	**26.11**	**6.21**	**29.00**	**30.21**	**1.20**
Original products	19.90	6.91	−13.00	29.00	26.42	−2.58
Generic products	0.00	19.20	19.20	0.00	3.78	3.78
**Policy-related varieties**	**100.00**	**100.00**	**0.00**	**100.00**	**100.00**	**0.00**
Original products	42.74	14.70	−28.04	80.08	80.49	0.41
Generic products	57.26	85.30	28.04	19.92	19.51	−0.41

*NAs, nucleos(t)ide analogs; DDDs, defined daily doses*.

### ITS Analysis of Changes in DDDs, Expenditures, and DDDc

Table 6 represented the results of the segmented linear analysis with ITS. The DDD of policy-related varieties has increased with the “4+7” policy by 639.56 thousand (*p* < 0.01). Under the “4+7” policy, the expenditures of policy-related varieties (trend coefficient: β_3_ = −349.97, *p* < 0.05) showed a decreasing trend. The DDDc of policy-related varieties had a substantial drop of ¥6.36 (*p* < 0.001) with the “4+7” policy, but the trend after the intervention has only decreased by ¥0.14 with no statistical significance.

**Table 6:** The result of ITSA analysis of hepatitis B antiviral NAs in Shenzhen.

**Categories**	**DDDs (thousands)**	**Expenditures (thousands yuan)**	**DDDc (yuan)**
	**Coef.(95%*C.I*.)**	**Coef.(95%*C.I*.)**	**Coef.(95%*C.I*.)**
**Policy-related varieties**			
Level change	639.56 (171.33, 1107.79)^**^	−4591.23 (−9361.61, 179.16)	−6.36 (−7.77, −4.95)^***^
Trend change	21.53 (−38.21, 81.27)	−349.97 (−694.66, −5.27)^*^	−0.14 (−0.30, 0.03)
Entecavir			
Level change	442.90 (74.97, 810.83)^*^	−3017.32 (−6437.40, 402.76)	−5.07 (−6.36, −3.77)^***^
Trend change	7.13 (−37.49, 51.76)	−264.99 (−528.05, −1.94)^*^	−0.10 (−0.23, 0.02)
Tenofovir Fumarate			
Level change	196.66 (81.62, 311.70)^**^	−1573.91 (−2956.38, −191.44)^*^	−11.40(−13.91, −8.89)^***^
Trend change	14.40 (−4.14, 32.94)	−84.97 (−183.00, 13.06)	−0.29 (−0.67, 0.10)
**Alternative varieties**			
Level change	−7.69 (−44.15, 28.78)	−149.03 (−632.70, 334.64)	−0.57 (−2.91, 1.78)
Trend change	0.01 (−4.95, 4.96)	−42.27 (−123.29, 38.75)	−0.30 (−0.59, 0.00)
Lamivudine			
Level change	−4.41 (−12.21, 3.38)	−49.48(−125.42, 26.46)	−0.53(−1.28, 0.23)
Trend change	0.43 (−0.58, 1.44)	3.38 (−6.29, 13.04)	−0.01 (−0.07, 0.05)
Adefovir dipivoxil			
Level change	−0.38 (−21.47, 20.71)	−48.58 (−162.50, 65.35)	−1.79(−5.30, 1.72)
Trend change	2.67 (−0.59, 5.92)	8.73 (−8.84, 26.30)	−0.09 (−0.53, 0.35)
Telbivudine			
Level change	−2.89 (−29.01, 23.22)	−50.97 (−510.56, 408.62)	0.02(0.00, 0.03)
Trend change	−3.09 (−7.56, 1.39)	−54.38 (−133.10, 24.35)	0.02 (0.01, 0.05)^***^
**Nucleotide analog**			
Level change	631.87 (140.83, 1122.92)^*^	−4740.26 (−9822.03, 341.52)	−6.17 (−7.43, −4.92)^***^
Trend change	21.54 (−39.88, 82.96)	−392.24 (−774.38, −10.09)^*^	−0.21 (−0.38, −0.04)^*^

*NAs, nucleos(t)ide analogs; DDDs, defined daily doses; DDDc, defined daily dose cost.*

* *p < 0.05,*

** *p < 0.01,*

*** *p < 0.05*.

The DDD of Entecavir has increased with the “4+7” policy (level coefficient: β_2_ = 442.90, *p* < 0.05). After the implementation of policy, the expenditures of Entecavir (trend coefficient: β_3_ = −264.99, *p* < 0.05) showed a decreasing trend. The DDDc of Entecavir had a substantial drop of ¥5.07 (*p* < 0.001) with the “4+7” policy. The DDD of Tenofovir Fumarate has increased with the “4+7” policy (level coefficient: β_2_ = 196.66, *p* < 0.01). After the implementation of the “4+7” policy, the expenditures of Entecavir (level coefficient: β_2_ = −1573.91, *p* < 0.05) showed a decreasing trend. The DDDc of Tenofovir Fumarate had a substantial drop of ¥11.40 (*p* < 0.001) with the “4+7” policy.

The post-intervention period witnessed a significant increase in the regression level for NA DDDs (level coefficient: β_2_ = 631.87, *p* < 0.05). The expenditures of NA (trend coefficient: β_3_ = 392.24, *p* < 0.05) showed a decreasing trend in the post-intervention period. After the intervention, there was a significant decrease in the regression slope and level for the DDDc of NA (level coefficient: β_2_ = −6.17, *p* < 0.001; trend coefficient: β_3_ = −0.21, *p* < 0.05).

Table 7 showed that the DDD of non-winning products in the policy-related varieties had a significant decrease in the regression slope and level (level coefficient: β_2_ = −621.81, *p* < 0.01; trend coefficient: β_3_ = −41.78, *p* < 0.05). The expenditures of non-winning products in the policy-related varieties also had a significant decrease in the regression slope and level (level coefficient: β_2_ = −5365.27, *p* < 0.05; trend coefficient: β_3_ = −388.68, *p* < 0.05). The DDDc of non-winning products in the policy-related varieties had a substantial increase of ¥4.37 (*p* < 0.001) after the implementation of the “4+7” policy.

**Table 7:** The result of ITSA analysis of hepatitis B antiviral NAs policy-related drugs in Shenzhen.

**Categories**	**DDDs (thousands)**	**Expenditures (thousands yuan)**	**DDDc (yuan)**
	**Coef.(95%*C.I*.)**	**Coef.(95%*C.I*.)**	**Coef.(95%*C.I*.)**
**Entecavir**			
Winning products			
Level change	–	–	0.62(0.62, 0.62)^***^
Trend change	–	–	–
Non-winning products			
Level change	−545.42 (−922.50, −168.34)^**^	−3630.08 (−7091.80, −168.37)^*^	7.90 (3.82, 11.99)^***^
Trend change	−37.75 (−63.62, −11.88)^**^	−292.82 (−554.35, −31.29)^*^	0.64(−0.09, 1.37)
**Tenofovir Fumarate**			
Winning products			
Level change	–	–	0.59 (0.59, 0.59)^***^
Trend change	–	–	–
Non-winning products			
Level change	−76.39 (−159.58, 6.80)	−1735.19 (−3126.28, −344.09)^*^	−0.07 (−1.91, −0.19)^*^
Trend change	−4.03 (−10.43, 2.38)	−95.85(−193.10, 1.39)	−0.45(−0.24, −0.50)^*^
**Policy-related varieties**			
Winning products			
Level change	–	–	0.61 (0.61, 0.61)^***^
Trend change	–	–	–
Non-winning products			
Level change	−621.81 (−1075.80, −167.82)^**^	−5365.27 (−10200.00, −546.36)^*^	4.37 (1.96, 6.78)^***^
Trend change	−41.78 (−72.18, −11.38)^**^	−388.68 (−732.17, −45.18)^*^	0.23 (−0.21, 0.68)

*NAs, nucleos(t)ide analogs; DDDs, defined daily doses; DDDc, defined daily dose cost.*

* *p < 0.05,*

** *p < 0.01,*

*** *p < 0.05*.

Table 8 revealed that the expenditures of original products and generic products both showed a decreasing trend in the post-intervention period (trend coefficient: β_3_ = −372.78, *p* < 0.05, trend coefficient: β_3_ = −130.78, *p* < 0.05, respectively). The DDDc of original products in the policy-related varieties was a significant decrease in the regression slope and level in Table 7 (level coefficient: β_2_ = −2.18, *p* < 0.05; trend coefficient: β_3_ = −0.32, *p* < 0.01).

**Table 8:** The result of ITSA analysis of hepatitis B antiviral NAs policy-related drugs in Shenzhen.

**Categories**	**DDDs (thousands)**	**Expenditures (thousands yuan)**	**DDDc (yuan)**
	**Coef. (95%*****C.I***.**)**	**Coef. (95%*****C.I***.**)**	**Coef. (95%*****C.I***.**)**
**Entecavir**			
Original products			
Level change	−49.03 (−136.13, 38.07)	−1792.36 (−4174.40, 589.67)	−2.75 (−4.76, −0.74)
Trend change	−9.49 (−20.82, 1.85)	−256.86 (−497.13, −16.59)^*^	−0.27 (−0.58, 0.05)
Generic products			
Level change	517.49 (200.05, 834.93)^**^	−1223.48(−2407.61, −39.34)^*^	−2.43 (−2.83, −2.03)^***^
Trend change	1.43 (−41.35, 44.20)	−141.66 (−228.12, −55.20)^**^	0.04 (−0.13, 0.00)^*^
**Tenofovir Fumarate**			
Original products			
Level change	−83.90(−169.68, 1.87)	−1856.81 (−3289.48, −424.14)^*^	−0.57 (−2.91, −0.19)^*^
Trend change	−5.21 (−11.99, 1.58)	−115.92 (−215.57, −16.27)^*^	−0.05 (−0.24, −0.10)^*^
Generic products			
Level change	–	–	–
Trend change	–	–	–
**Policy-related varieties**			
Original products			
Level change	−132.93 (−302.42, 36.55)	−3649.17 (−7428.38, 130.03)	−2.18 (−3.83, −0.53)^*^
Trend change	−14.69(−31.37, 1.99)	−372.78(−691.28, −54.28)^*^	−0.32 (−0.54, −0.09)^**^
Generic products			
Level change	790.54 (440.27, 1140.81)^***^	−1062.20 (−2230.25, 105.86)	−2.56 (−3.14, −1.98)^***^
Trend change	19.85 (−33.17, 72.87)	−130.78 (−215.06, −46.49)^**^	−0.06(−0.15, 0.03)

*NAs, nucleos(t)ide analogs; DDDs, defined daily doses; DDDc, defined daily dose cost.*

* *p < 0.05,*

** *p < 0.01,*

*** *p < 0.05*.

## Discussion

If the economies of scale exist in the group purchasing of drugs, the scale of the “4+7” pilot policy should affect the bargaining power, because the larger the purchase of drugs in a larger volume, the closer it is to group purchasing. The National Centralized Drug Purchasing and Using Pilot Program also can be regarded as group purchasing which had bargaining power in drug purchasing (54).

Medicine affordability was attained by price control (55). Pharmaceutical price control measures are the mechanism used by low- and middle-income countries to keep drug prices in check while increasing affordability. After decreasing the price of drugs, the accessibility may be improved. This study explored the impacts of the “4+7” pilot policy on the volume, expenditures, daily cost, and distribution structure of NAs in Shenzhen city. Price is the primary determinant of drug affordability. After the implementation of the “4+7” policy, the DDDc of NAs decreased by 69.08%. Especially, the DDDc of policy-related drugs decreased by 73.26%, the DDDc of Entecavir and Tenofovir Fumarate decreased by 71.50 and 78.77%, respectively. And the results of the segmented linear analysis with ITS also showed that the DDDc of NA, policy-related varieties, and Entecavir and Tenofovir Fumarate were all significantly dropped in the level (*p* < 0.001). These findings indicated that the “4+7” policy played a certain price reduction effect on NA, especially policy-related varieties such as Entecavir and Tenofovir Fumarate. These showed that the “4+7” policy reduced the price of medicines. The price of NAs was decreasing, the burden of medication for patients with CHB was lightened. The implementation of the “4+7” policy has effectively reduced the medication burden of some patients (56).

After decreasing the price of drugs, the accessibility was improved. After the implementation of the “4+7” policy, the volume of NAs was increased significantly. Especially, the volume of policy-related drugs was increased by 95.60%, the volume of Entecavir and Tenofovir Fumarate was increased by 80.45 and 156.58%, respectively. And the results of the segmented linear analysis with ITS also showed that the volume of NA, policy-related varieties, and Entecavir and Tenofovir Fumarate was significantly increased in the level (*p* < 0.05). The “4+7” policy has raised the volume of Entecavir and Tenofovir Fumarate. The proportion of Entecavir and Tenofovir Fumarate in NAs DDDs was increased. It was consistent with the result of Sheng Liangliang's study which analyzed the hospital's application of nucleoside anti-hepatitis B drugs based on “4+7” quantified purchasing in Dalian (57). Dalian is also 1 of 11 pilot cities. The results indicated that more patients with CHB received standard antiviral therapy.

The implementation of the “4+7” policy has effectively improved the use efficiency of medical insurance funds (56). The expenditures of NAs were dropped by 45.43% during the post-intervention period, among these, policy-related drugs were dropped by 47.69%. And the results of the segmented linear analysis with ITS also showed that the expenditure of NAs was significantly decreased in the trend (*p* < 0.05).

The main causes of growth in pharmaceutical expenditure during the last decades are volume growth vs. price growth on the one hand and the release of the medical needs of the patient on the other hand (58). In this study, we found that the volume of policy-related drugs was increased by 95.60%, and the expenditures of policy-related drugs were dropped by 47.69%. It indicated that the “4+7” policy plays a good role in controlling pharmaceutical expenditure.

The DDDc of winning products in policy-related varieties was low. The doctors guide patients to choose winning products, which were high quality and low cost. The results also showed that the proportion of winning products was increased by 83.42 percent point. The consistency evaluation of generic drugs has made significant progress. These generic drugs have reached the same level of quality and efficacy as the original drugs (59).

According to the previous studies, in addition to price control, one of the most commonly used policies and strategies to improve medicine affordability and accessibility is the promotion of generic medicine use (60). Most of the Association of Southeast Asian Nations (ASEAN) countries, especially Malaysia and Indonesia, applied generic medicine promotion to enhance the use of generic medicines as they are much cheaper (61). The proportion of generic products in policy-related varieties is growing by 28.04 percent point and the expenditure of generic products is decreased by 48.77% accordingly in this study.

In China, all generic drugs participating in the “4+7” pilot policy have been evaluated for consistency in quality and efficacy. The consistency evaluation of generic drugs has made significant progress. These generic drugs have reached the same level of quality and efficacy as the original drugs (59). The patients were inclined to choose high-quality generic products because of the high reimbursement rate (56). After the implementation of the “4+7” policy, the DDDs of generic products in policy-related varieties were increased by 191.37%. The expenditures and DDDc of generic products in policy-related varieties were decreased by 48.77, 82.42% respectively.

In the management of drug use, the hospital has strengthened policy guidance and supervision, such as decomposing indicators to individual departments and formulating certain system specifications. The doctors guide patients to choose winning products, which were high quality and low cost. The “4+7” policy given priority to using winning products, which has promoted the replacement of winning products with non-winning products (62). The proportion of non-winning products in policy-related varieties is decreased by 83.42 percent points.

The availability of generics could lead to a reduction in prices for brand-name drugs and thereby contribute to savings in pharmaceutical expenditures (63). This study revealed that the DDDc of original products showed a decrease in the post-intervention period (trend coefficient: β_2_ = −2.56, *p* < 0.001).

The present study had some limitations that should be borne in mind when interpreting the results. One of the limitations was the lack of inclusion of drugstores, as one of the main stakeholders of the pharmaceutical industry in the study. Because only public medical institutions were included in the purchasing alliance in the “4+7” pilot policy. We did not analyze the tendency of drug use volume, expenditures, and DDDc of NAs in the drugstore. However, antiviral treatment of chronic hepatitis B (CHB) need to take medicine for a long time. Reimbursement policy was the most important factor that was affecting the antiviral treatment. And patients with CHB have Outpatient Chronic Disease Insurance, which has a reimbursement rate for medical expenditures. Second, we only used 24 months of time series data for ITS analysis and only 8 months of time series data in the post-intervention. In exploring the long-term trend of the “4+7” pilot policy, it was better if we can get 12 months of time series data in the post-intervention. We will get total pilot cities as research objects and full purchasing cycle as research time points in the further study. The main strength of this study was to using ITS quantitative analysis in the impact of the “4+7” pilot policy. It may be a valuable reference for policy effect evaluation. It offered suggestions for policy promotion.

## Conclusion

After the implementation of the “4+7” policy, the DDDc of NAs decreased, the accessibility of policy-related drugs was improved, and the usage of generic medicine was promoted.

## Data Availability Statement

The original contributions presented in the study are included in the article/supplementary material, further inquiries can be directed to the corresponding author/s.

## Ethics Statement

This study was approved by the Institutional Review Board of Wuhan University. Patients or public were not involved in this study.

## Author Contributions

ZM, DC, and XW: conceptualization and project administration. XW: formal analysis and writing—original draft. ZM and DC: funding acquisition and supervision. XW, SY, LM, ZL, ZY, XG, YL, and YY: investigation. XW and YY: methodology. XW, LC, SY, and YY: writing—review and editing. All authors have read and agreed to the published version of the manuscript.

## Funding

This study was supported by the Global Health Institute, Wuhan University, China.

## Conflict of Interest

The authors declare that the research was conducted in the absence of any commercial or financial relationships that could be construed as a potential conflict of interest.

## Publisher's Note

All claims expressed in this article are solely those of the authors and do not necessarily represent those of their affiliated organizations, or those of the publisher, the editors and the reviewers. Any product that may be evaluated in this article, or claim that may be made by its manufacturer, is not guaranteed or endorsed by the publisher.
